# Determining post-treatment surveillance criteria for predicting the elimination of *Schistosoma mansoni* transmission

**DOI:** 10.1186/s13071-019-3611-8

**Published:** 2019-09-16

**Authors:** Jaspreet Toor, James E. Truscott, Marleen Werkman, Hugo C. Turner, Anna E. Phillips, Charles H. King, Graham F. Medley, Roy M. Anderson

**Affiliations:** 1 London Centre for Neglected Tropical Disease Research, Department of Infectious Disease Epidemiology, St Mary’s Campus, Imperial College London, Norfolk Place, London, W2 1PG UK; 2 MRC Centre for Global Infectious Disease Analysis, Department of Infectious Disease Epidemiology, School of Public Health, Faculty of Medicine, St Mary’s Campus, Imperial College London, Norfolk Place, London, W2 1PG UK; 3 The DeWorm3 Project, The Natural History Museum of London, London, SW7 5BD UK; 4 Oxford University Clinical Research Unit, Wellcome Trust Major Overseas Programme, Ho Chi Minh City, Vietnam; 5 Centre for Tropical Medicine and Global Health, Nuffield Department of Medicine, University of Oxford, Oxford, UK; 6 Center for Global Health and Diseases and Department of Mathematics, Case Western Reserve University, 10900 Euclid Avenue LC: 4983, Cleveland, OH 44106 USA; 7 Centre for Mathematical Modelling of Infectious Disease, London School of Hygiene and Tropical Medicine, London, UK

**Keywords:** Schistosomiasis, Elimination of transmission, Stochastic individual-based model, Positive predictive value, Prevalence threshold, Post-treatment surveillance, Monitoring and evaluation

## Abstract

**Background:**

The World Health Organization (WHO) has set elimination (interruption of transmission) as an end goal for schistosomiasis. However, there is currently little guidance on the monitoring and evaluation strategy required once very low prevalence levels have been reached to determine whether elimination or resurgence of the disease will occur after stopping mass drug administration (MDA) treatment.

**Methods:**

We employ a stochastic individual-based model of *Schistosoma mansoni* transmission and MDA impact to determine a prevalence threshold, i.e. prevalence of infection, which can be used to determine whether elimination or resurgence will occur after stopping treatment with a given probability. Simulations are run for treatment programmes with varying probabilities of achieving elimination and for settings where adults harbour low to high burdens of infection. Prevalence is measured based on using a single Kato-Katz on two samples per individual. We calculate positive predictive values (PPV) using PPV ≥ 0.9 as a reliable measure corresponding to ≥ 90% certainty of elimination. We analyse when post-treatment surveillance should be carried out to predict elimination. We also determine the number of individuals across a single community (of 500–1000 individuals) that should be sampled to predict elimination.

**Results:**

We find that a prevalence threshold of 1% by single Kato-Katz on two samples per individual is optimal for predicting elimination at two years (or later) after the last round of MDA using a sample size of 200 individuals across the entire community (from all ages). This holds regardless of whether the adults have a low or high burden of infection relative to school-aged children.

**Conclusions:**

Using a prevalence threshold of 0.5% is sufficient for surveillance six months after the last round of MDA. However, as such a low prevalence can be difficult to measure in the field using Kato-Katz, we recommend using 1% two years after the last round of MDA. Higher prevalence thresholds of 2% or 5% can be used but require waiting over four years for post-treatment surveillance. Although, for treatment programmes where elimination is highly likely, these higher thresholds could be used sooner. Additionally, switching to more sensitive diagnostic techniques, will allow for a higher prevalence threshold to be employed.

**Electronic supplementary material:**

The online version of this article (10.1186/s13071-019-3611-8) contains supplementary material, which is available to authorized users.

## Background

Schistosomiasis is an intestinal or urogenital neglected tropical disease (NTD) caused predominantly by infection with *Schistosoma mansoni*, *S. haematobium* or *S. japonicum*. Over 200 million people require preventive chemotherapy (PC) for the disease across 52 endemic countries [1]. As school-aged children (SAC; 5–14 years of age) are most likely to be infected by *Schistosoma* species, PC using mass drug administration (MDA) of praziquantel has focused on this age group. By 2020, the World Health Organization (WHO) aims to increase coverage such that 75% of SAC at risk will be regularly treated in endemic countries [2]. Adults are also likely to be infected and in areas of high transmission, WHO guidelines recommend treatment of adults at risk [3]. Recent modelling work has highlighted the importance of including adults within treatment programmes, with coverage levels impacted by the burden of infection in adults relative to SAC, particularly in high prevalence (transmission) settings [4, 5]. Pre-school aged children (pre-SAC) are not presently eligible for treatment with praziquantel [6]. However, recent work shows that praziquantel may be used on an individual diagnosis level to treat pre-SAC, provided the dosage is correct [7].

The WHO has set goals of morbidity control and elimination as a public health problem, defined by reaching < 5% and < 1% prevalence of heavy-intensity infections (eggs per gram ≥ 400) in SAC, respectively [3]. These goals are to be achieved using MDA with the treatment frequency determined by the prevalence prior to treatment, as recommended by the WHO [3]. Once prevalence of infection is less than 1% by Kato-Katz among SAC, the WHO currently recommends conducting serology once every two years and PC is then stopped if this is negative. The end goal for schistosomiasis has been set as elimination (interruption of transmission) to be reached by 2025 in the Region of the Americas, the Eastern Mediterranean Region, the European Region, the South-East Asia Region and the Western Pacific Region, and in selected countries of the African Region [3]. This is achieved by reducing the incidence of infection to zero [3]. Currently, there is a lack of appropriate guidance on how to determine whether elimination has occurred, as well as how to identify potential resurgence (bounce-back) after stopping treatment once very low prevalence levels have been reached. Hence, it is important that the appropriate protocols, based on understanding of transmission dynamics, are designed to determine the elimination criteria for schistosomiasis treatment. It is important to note that the WHO treatment guidelines and the 2030 WHO goals are currently under review.

As schistosome parasites reproduce sexually within the human host, both sexes need to be present within an individual host to produce fertilized eggs (maintaining the transmission cycle). As the prevalence of infection declines, the likelihood of having both sexes present in the same individual declines. This results in a breakpoint of transmission where below a critical prevalence threshold, the parasites cannot reproduce frequently enough to maintain transmission leading to eradication of infection, even without ongoing treatment in the absence of frequent immigration of infected individuals into a defined area. The optimal prevalence threshold has been defined for other helminth infections, such as the soil-transmitted helminths [8, 9]. Here we apply similar methods for determining the prevalence threshold for *S. mansoni*. Notably, the lifecycle of schistosomes includes complexities, such as asexual reproduction within the intermediate snail host, which are accounted for in the model.

In this study, we provide guidance on the post-treatment surveillance criteria for *S. mansoni* in terms of the prevalence threshold that is required to reliably predict elimination, the number of individuals that need to be sampled within a community, and how long after the last round of treatment this should be checked.

## Methods

We employed a stochastic individual-based mathematical model to define the prevalence threshold, i.e. prevalence of infection, which needs to be reached to ensure that elimination will be achieved with defined probability. The model tracks individuals within the population (both human hosts and their parasite populations) as they become infected as well as treated over time. The model has been previously used for the soil-transmitted helminths [9, 10] and has been adapted to represent *S. mansoni* transmission (parameter values in Table 1). The mean value of the stochastic simulations aligns with the predictions of an age-structured partial differential equation deterministic model [11]. Within the model we focused on a single community without migration. We measured prevalence using a single Kato-Katz on two separate stool samples per individual as the diagnostic test. We used two age-profiles of infection with low and high burdens of infection in adults relative to SAC (produced by varying the age specific contact rates) and varying associated transmission intensities (i.e. basic reproductive (R_0_) values; Table 1) [5, 12].

**Table 1:** Parameter values used for *Schistosoma mansoni*

Parameter	Value	Source
Fecundity (egg output per female worm in absence of density dependence)	0.34 eggs/female worm/sample	[11, 23, 24]
Variation in egg counts within individuals	0.87	[23, 24]
Aggregation parameter for high baseline prevalence settings	0.24	[5, 25, 26]
Density dependence fecundity	0.0007/female worm	[12, 25]
Worm lifespan	5.7 years	[11, 27]
Drug efficacy	86%	[28]
Low adult burden setting: age-specific contact rates for 0–5, 5–10, 10–16, 16+ years of age	0.01, 1.2, 1, 0.02	[5, 12]
High adult burden setting: age-specific contact rates for 0–5, 5–12, 12–20, 20+ years of age	0.01, 0.61, 1, 0.12	[5, 12]
Prevalence of infection	Percentage of population having egg count threshold (or eggs per gram, epg) > 0	–
Prevalence of heavy-intensity infections	Percentage of population having egg count threshold ≥ 16 (epg ≥ 400 divided by 24 to convert to egg count)	[29]
Human demography	Based on Uganda’s demographic profile	[30, 31]

We simulated high baseline prevalence settings (≥ 50% SAC prevalence by Kato-Katz) and carried out annual treatment at high coverage levels (85% SAC + 40% adults and 100% SAC + 100% adults) in order to reduce prevalence to very low levels within 8 to 12 years. We assumed treatment coverage occurs at random at each round of MDA and that there is no systematic non-adherence. Notably, annual treatment of 75% SAC-only for 15 years did not achieve elimination in any of these high prevalence settings. 1000 model iterations were run for each scenario and the model was pre-run for 10 years to achieve a stable equilibrium prior to MDA. Elimination was then checked at year 60 (50 years after MDA initiated). The scenarios vary from low to high likelihoods of elimination occurring. Scenarios where elimination was highly unlikely (≤ 13%) or likely (≥ 89%) were not focused on as they were not informative for this analysis (scenarios shown in Table 2 and Additional file 1: Figure S1).

**Table 2:** Settings and treatment strategies used within the model simulations showing the likelihood of achieving elimination. Settings in non-bold text were not focused on in the analysis due to very low/high likelihood of achieving elimination. Mean baseline prevalence is shown for across the entire community (all ages). Corresponding age-specific contact rates for the low and high adult burden settings are shown in Table 1

Setting	Annual treatment strategy	Programme length (years)	Simulations achieving elimination (%)
Low adult burden (R_0_ = 3; mean baseline prevalence 58%)	75% SAC-only	15	0
	**85% SAC** + **40% adults**	10	13
		**12**	**45**
		15	89
	**100% SAC** + **100% adults**	5	0
		**8**	**60**
		10	99
		15	100
High adult burden (R_0_ = 4; mean baseline prevalence 61%)	75% SAC-only	15	0
	85% SAC + 40% adults	15	0
	**100% SAC** + **100% adults**	**10**	**60**
		15	99

We determined the prevalence threshold that needs to be reached to distinguish between achievement of elimination or bounce-back by calculating positive/negative predictive values (PPV/NPV). The PPV is the proportion of eliminations detected by the threshold statistic that result in long-term eliminations, whereas, the NPV is the proportion of bounce-backs detected by the threshold statistic that result in resurgence of the disease. The threshold statistic for this analysis is based on prevalence. In order to reliably predict eliminations, a high PPV is required. A PPV of 1 is ideal as this corresponds to 100% certainty of elimination. Here we regarded PPV ≥ 0.9 as a reliable measure corresponding to ≥ 90% certainty of elimination (therefore regarding PPV < 0.9 as an unreliable measure corresponding to < 90% certainty of elimination). PPV and NPV were calculated for Kato-Katz prevalence threshold values of 0.5, 1, 2 and 5% up to 12 years after the last round of MDA to determine the appropriate timepoint for post-treatment surveillance (PPV and NPV shown in Additional file 1: Table S1). Additionally, we tested these Kato-Katz prevalence threshold values for scenarios with low to high likelihoods of elimination occurring.

We also determined the sample sizes (whilst sampling from the entire community across all age groups at random) required to predict whether elimination has been achieved. For our single community analysis, we sampled between 100 individuals up to the entire population (where the population size was set at 500 or 1000 individuals).

## Results

**Fig. 1:**
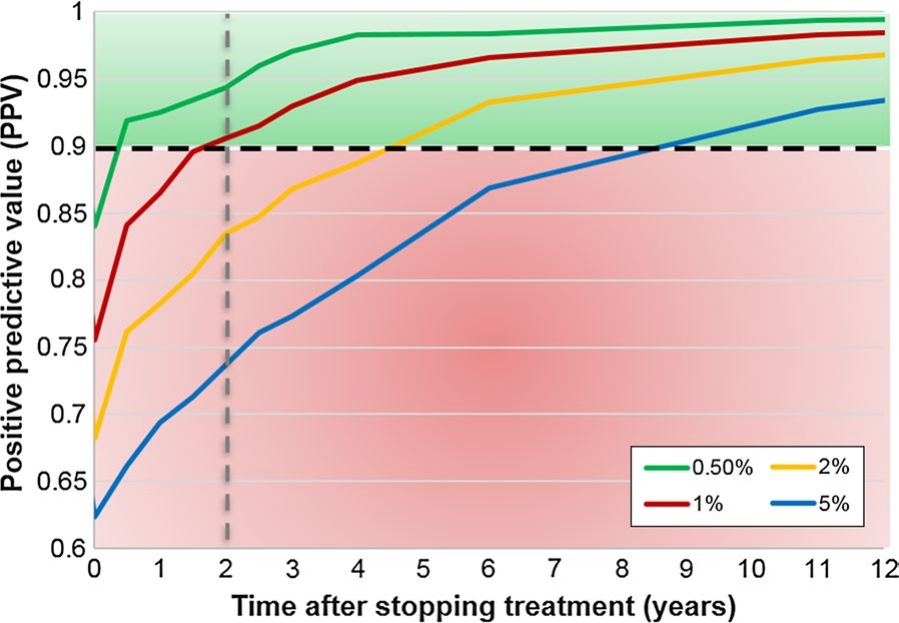
Positive predictive values (PPV) over time for varying Kato-Katz prevalence threshold values (0.5, 1, 2 and 5%) whilst sampling 200 individuals across the entire community (population size is set at 500). The trends are for the high adult burden setting where treatment has been carried out for 100% school-aged children and 100% adults annually for 10 years. The dashed black line is where the PPV is 0.9 and the grey line is where the time after stopping treatment is 2 years. The area shaded in red is where PPV < 0.9 and in green is where PPV ≥ 0.9. Corresponding PPV and negative predictive values (NPV) shown in Additional file 1: Table S1

A prevalence threshold of 0.5% by Kato-Katz is most sufficient for predicting elimination six months after stopping treatment with a PPV ≥ 0.9. A prevalence threshold of 1% can predict elimination at least two years after the last round of treatment (PPV ≥ 0.9). Higher prevalence threshold values of 2% or 5% require waiting over four years for post-treatment surveillance (Fig. 1). These prevalence threshold measures are representative of prevalence across the entire community (not SAC-only).

**Fig. 2:**
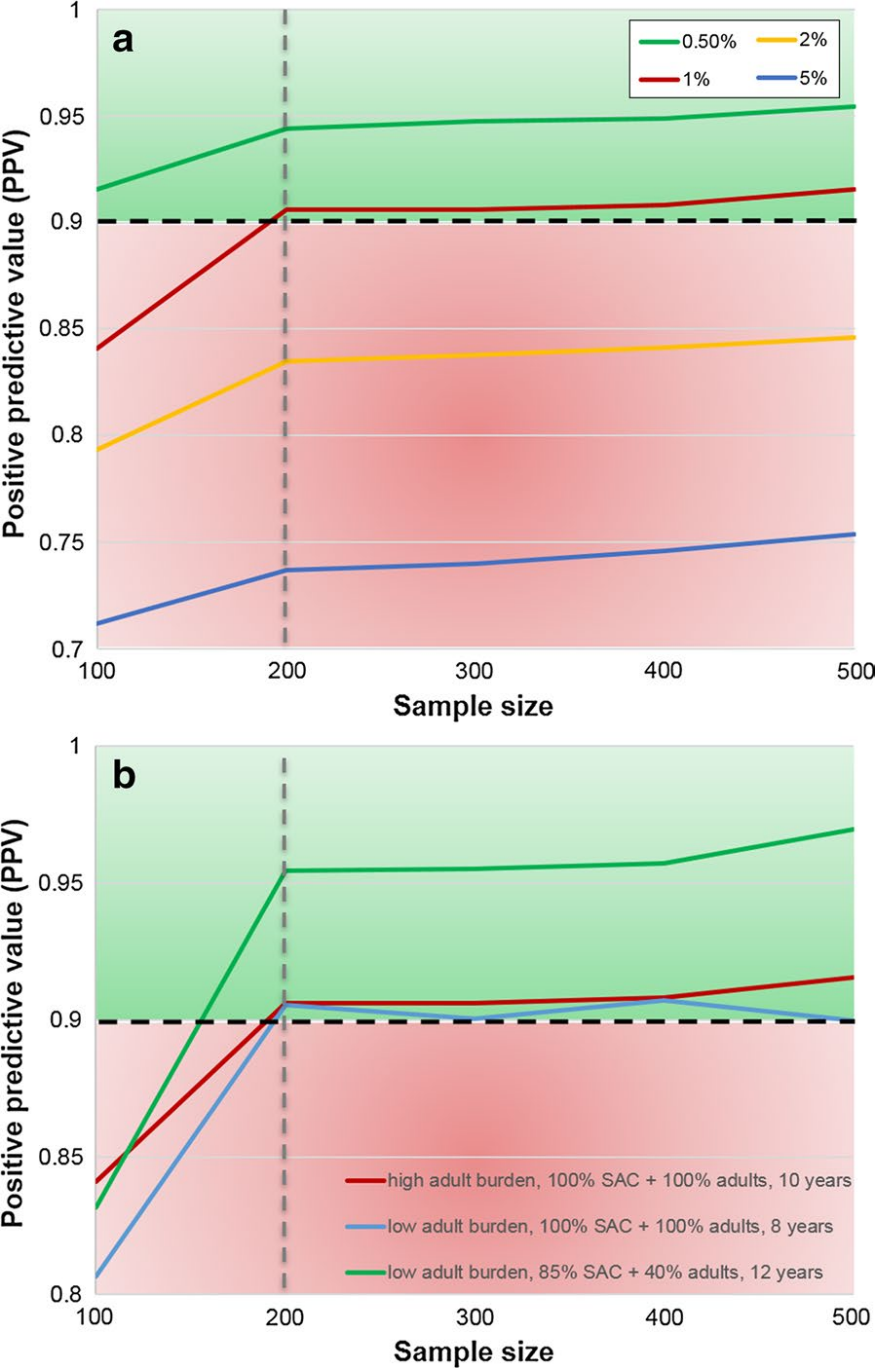
Positive predictive values (PPV) for varying sample sizes of 100 to 500 individuals across the entire community (population size is set at 500). **a** For high adult burden setting using 0.5 to 5% prevalence threshold values 2 years post-treatment. **b** For three scenarios using a 1% prevalence threshold value 2 years post-treatment. In **a** and **b** the dashed black line is where the PPV is 0.9 and the grey line is where the sample size is 200. The area shaded in red is where PPV < 0.9 and in green is where PPV ≥ 0.9

When using a 0.5% prevalence threshold, for a population of size 500, it is sufficient to sample 100 individuals across all age groups two years post-treatment (Fig. 2a). However, when using a prevalence threshold of 1%, a sample size of 100 individuals is not informative as at least 200 individuals need to be sampled to achieve a PPV ≥ 0.9 (Fig. 2a, b). Similarly, a sample size of 200 individuals was reliable for larger population sizes of 1000 individuals. These results hold regardless of whether there is a low or high adult burden of infection. Prevalence thresholds of 2% and 5% are not sufficient two years post-treatment as they do not achieve PPV ≥ 0.9 even if the entire population is sampled (Fig. 2a), we would need to wait over four years post-treatment for these thresholds to be informative (Fig. 1).

**Fig. 3:**
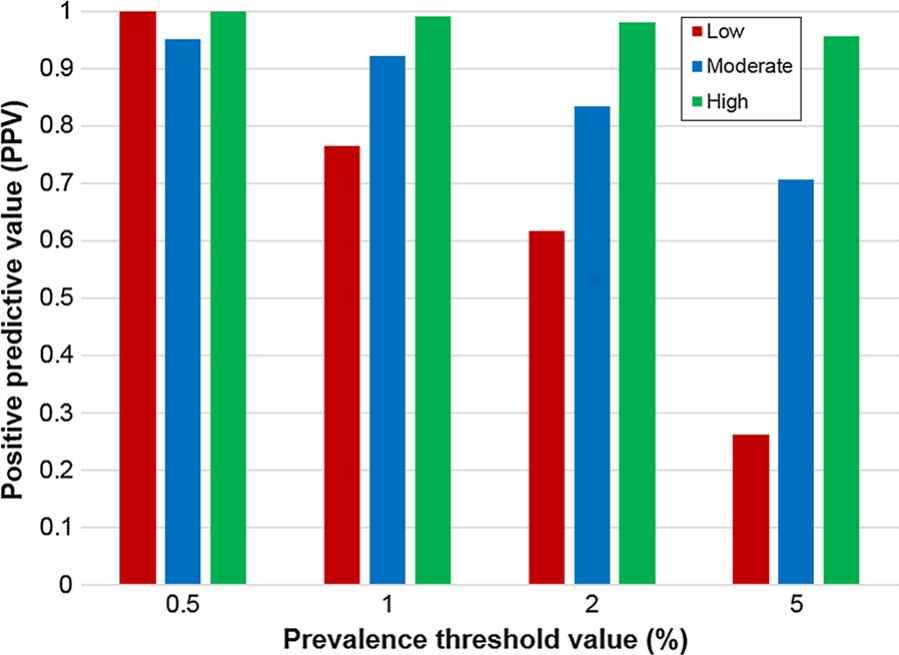
Prevalence threshold value and positive predictive values (PPV) for treatment programmes with low (13%), moderate (45–60%) and high (91%) likelihoods of achieving elimination. Values are shown for surveillance occurring 2 years post-treatment with a sample size of 200 individuals (population size is set at 500)

The required prevalence threshold can be adapted depending on the likelihood of achieving elimination. For treatment programmes which are highly likely to achieve elimination (i.e. programmes which have maintained high coverage and adherence over each round of MDA), a higher prevalence threshold can be used, for example, in scenarios where ≥ 90% scenarios reach elimination, a threshold of 5% is sufficient to achieve a high PPV value (Fig. 3). For treatment programmes which are very unlikely to achieve elimination, a smaller threshold of 0.5% is required to achieve a high PPV value (Fig. 3). In the simulations presented in this paper, we have focused on scenarios of different R_0_ values and MDA coverage where there is a moderate likelihood of elimination (45–60%; Table 2). In these cases, a prevalence threshold of 0.5 or 1% two years post-treatment gives a reliable PPV greater than 0.9 (Fig. 3).

## Discussion

**Fig. 4:**
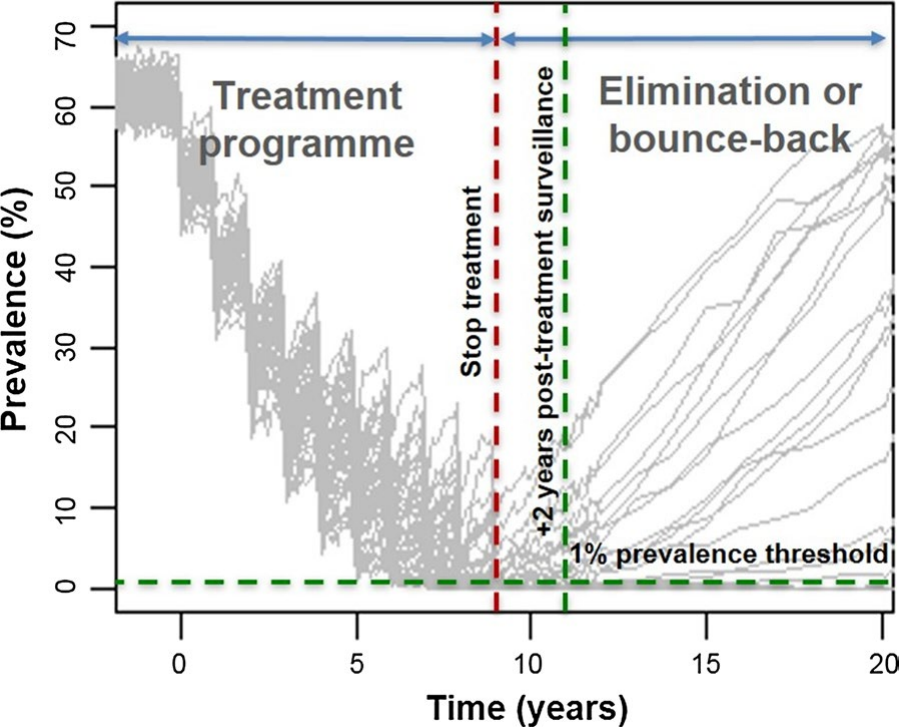
Simulations achieving elimination or bounce-back after stopping treatment (50 simulations are shown for a total population size of 500 individuals) for a high adult burden setting; treating 100% SAC + 100% adults annually for 10 years (10 rounds of treatment starting at year 0 and ending at year 9). Model recommendations are shown in green dashed lines where post-treatment surveillance is carried out 2 years after the last round of treatment using a 1% prevalence threshold

Although the WHO has set elimination as the end goal for schistosomiasis [3], there has been a lack of guidance on the criteria required for determining whether elimination or resurgence will occur after stopping treatment. Currently there is little guidance for programme managers on what to do once very low levels of prevalence have been reached. Using our modelling approach, we have shown that a prevalence threshold of 1% by Kato-Katz and a sample size of 200 individuals (in a defined community of 500 to 1000 individuals) is sufficient for predicting *S. mansoni* elimination two years after cessation of treatment (Fig. 4).

### Programmatic limitations

A prevalence threshold of 0.5% is sufficient to predict elimination six months after stopping treatment. However, this can be difficult to measure reliably using Kato-Katz in the field as it is a very low prevalence. Higher prevalence thresholds of 2% or 5% can be used but require waiting for over four years for post-treatment surveillance which may not be ideal for treatment programmes. Hence, due to such programmatic limitations, we recommend using a 1% prevalence threshold two years after stopping treatment.

A higher prevalence threshold or a smaller sample size could be used with lower accuracy (PPV < 0.9 i.e. less than 90% certainty of elimination). This approach may be a viable option for treatment programmes which are highly certain that elimination will be achieved due to consistently high MDA coverage and individual adherence to treatment over many rounds of MDA. However, to accurately determine achievement of elimination with greater than 90% certainty, we recommend a 1% prevalence threshold with a sample size of 200 individuals.

As we have focused on optimizing the PPV (PPV ≥ 0.9), rather than the NPV, we are more accurately identifying elimination rather than resurgence. Alternatively, the analysis could be used to optimize NPV if predicting resurgence is the aim. Ideally, both PPV and NPV should be ≥ 0.9, and for our recommendations this holds (PPV and NPV shown in Additional file 1: Table S1).

Within our high baseline prevalence simulations, high coverage levels such as 100% SAC and 100% adult treatment were used in order to reduce prevalence to very low levels within 12 years. Lower coverage levels would require a longer MDA programme. In contrast, communities with a lower baseline prevalence would likely require lower coverage levels or a shorter MDA programme to achieve elimination.

### Model limitations and future work

As programmes move from morbidity control towards elimination, diagnostic techniques are becoming increasingly important as prevalence needs to be measured at low levels. It is important to consider which diagnostic techniques will be used in monitoring schistosomiasis infection. The traditional Kato-Katz diagnostic (currently recommended diagnostic by WHO [13]) has low sensitivity to detect infection at very low intensities and prevalences [14]. However, the point-of-care circulating cathodic antigen (POC-CCA) diagnostic technique performs better at detecting infection at low prevalence levels due to increased sensitivity [15–17]. Within this analysis, we have used Kato-Katz as the diagnostic to measure prevalence. However, a more sensitive diagnostic test, such as POC-CCA, or using more than two Kato-Katz will likely allow for a higher prevalence threshold [18]. Future work will investigate how prevalence threshold and sample sizes vary for such diagnostics. Whilst considering diagnostics within monitoring and evaluation activities, the economic costs also need to be considered [19].

Within our analysis, the sampling has been carried out at random across the community from all age groups. A sample size of 200 individuals has proven to be informative for settings where adults harbor both low and high burdens of infection. However, this could be adapted to sampling from specific age groups, e.g. SAC-only or SAC and adults, as this may reveal that sampling from adults is more important in regions where adults are highly infected. Previous work has shown that monitoring SAC and adults is important for determining appropriate treatment strategies, particularly in high prevalence settings [5].

Schistosomiasis is a focal disease as prevalence levels have been shown to vary widely between communities on a variety of spatial scales. District-level mapping for estimating schistosomiasis prevalence has shown that sampling less children in more schools rather than more children in less schools increases accuracy of prevalence estimates whilst optimizing cost-efficiency [20]. Our analysis has focused on a single community with population sizes of 500 to 1000 individuals, thereby assuming no immigration of infected individuals from neighboring communities where infection may persist. Future analyses will be extended to simulate multiple communities to capture the impact of spatial heterogeneity and migration. Furthermore, analyses will be extended to other schistosome species, such as *S. haematobium*, as well as the incorporation of risks posed by emerging widespread zoonotic schistosome species [21, 22].

## Conclusions

We have found that a prevalence threshold value of 1% by Kato-Katz is optimal (ensuring PPV ≥ 0.9 i.e. ≥ 90% certainty) for predicting *S. mansoni* elimination at least two years after the last round of treatment using a sample size of 200 individuals (where the total population size is 500 to 1000 individuals). We hope this study provides clear guidance on the post-treatment surveillance which needs to be carried out when approaching elimination for schistosomiasis in a defined area.

## Additional file

**Additional file 1: Figure S1.** Simulations achieving elimination or resurgence after stopping mass drug administration (50 simulations are shown for each scenario). **a** Low adult burden setting; treating 85% SAC + 40% adults annually for 12 years. **b** Low adult burden setting; treating 100% SAC + 100% adults annually for 8 years. **c** High adult burden setting; treating 100% SAC + 100% adults annually for 10 years. **Table S1.** Positive and negative predictive values (PPV and NPV) whilst sampling 200 individuals across the entire community (population size is 500) using single Kato-Katz on two samples per individual. Values are shown for high adult burden setting where treatment has been carried out for 100% school-aged children and 100% adults annually for 10 years. For each prevalence threshold, values highlighted in blue are time points for which PPV ≥ 0.9 and in grey are time points for which PPV < 0.9. PPV shown in Fig. 1.

## Data Availability

The datasets generated and/or analysed during the present study are not publicly available due to the large number of model iterations run for each scenario but are available from the corresponding author on reasonable request. All other data used during this study are included in the cited sources.

## Abbreviations

WHO: World Health Organization
MDA: mass drug administration
NTD: neglected tropical disease
PC: preventive chemotherapy
SAC: school-aged children
Pre-SAC: pre-school aged children
PPV: positive predictive value
NPV: negative predictive value
