# Supplementary material for: Determining post-treatment surveillance criteria for predicting the elimination of Schistosoma mansoni transmission
Source: Parasit Vectors. 2019 Sep 16;12:437. doi: 10.1186/s13071-019-3611-8 (PMC6745786; doi:10.1186/s13071-019-3611-8)
Supplement: Supplementary file 1 — Additional file 1: Figure S1. Simulations achieving elimination or resurgence after stopping mass drug administration (50 simulations are shown for each scenario). a Low adult burden setting; treating 85% SAC + 40% adults annually for 12 years. b Low adult burden setting; treating 100% SAC + 100% adults annually for 8 years. c High adult burden setting; treating 100% SAC + 100% adults annually for 10 years. Table S1. Positive and negative predictive values (PPV and NPV) whilst sampling 200 individuals across the entire community (population size is 500) using single Kato-Katz on two samples per individual. Values are shown for high adult burden setting where treatment has been carried out for 100% school-aged children and 100% adults annually for 10 years. For each prevalence threshold, values highlighted in blue are time points for which PPV ≥ 0.9 and in grey are time points for which PPV < 0.9. PPV shown in Fig. 1. [file 13071_2019_3611_MOESM1_ESM.docx]

**Additional file 1**


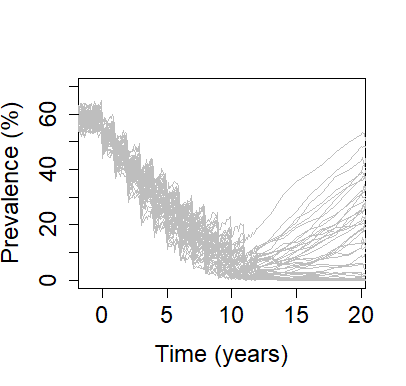


**a**


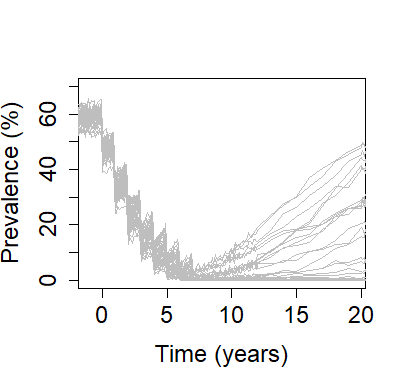

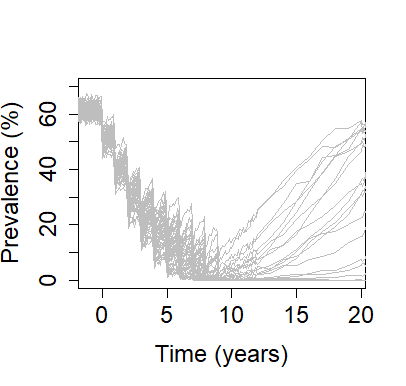


**c**

**b**

**Figure S1:** Simulations achieving elimination or resurgence after stopping mass drug administration (50 simulations are shown for each scenario). **a** Low adult burden setting; treating 85% SAC + 40% adults annually for 12 years. **b** Low adult burden setting; treating 100% SAC + 100% adults annually for 8 years. **c** High adult burden setting; treating 100% SAC + 100% adults annually for 10 years.

**Table S1:** Positive and negative predictive values (PPV and NPV) whilst sampling 200 individuals across the entire community (population size is 500) using single Kato-Katz on two samples per individual. Values are shown for high adult burden setting where treatment has been carried out for 100% school-aged children and 100% adults annually for 10 years. For each prevalence threshold, values highlighted in blue are time points for which PPV ≥ 0.9 and in grey are time points for which PPV < 0.9. PPV shown in Fig. 1.

| **Time after stopping treatment (years)** | **PPV/NPV for prevalence threshold value (prevalence of infection by Kato-Katz; %)** | | | |
| --- | --- | --- | --- | --- |
|  | **0.5** | **1** | **2** | **5** |
| 0 | 0.840/0.885 | 0.756/0.981 | 0.682/1 | 0.623/1 |
| 0.5 | 0.919/0.867 | 0.841/0.986 | 0.762/1 | 0.661/1 |
| 1 | 0.925/0.865 | 0.865/0.957 | 0.782/1 | 0.693/1 |
| 1.5 | 0.934/0.863 | 0.896/0.971 | 0.804/1 | 0.713/1 |
| 2 | 0.944/0.898 | 0.906/0.953 | 0.835/1 | 0.737/1 |
| 2.5 | 0.960/0.901 | 0.915/0.984 | 0.848/1 | 0.761/1 |
| 3 | 0.970/0.896 | 0.930/0.977 | 0.868/1 | 0.773/1 |
| 4 | 0.983/0.907 | 0.949/0.973 | 0.887/0.994 | 0.803/1 |
| 6 | 0.984/0.935 | 0.966/0.978 | 0.933/1 | 0.869/1 |
| 11 | 0.993/0.953 | 0.983/0.984 | 0.965/1 | 0.928/1 |
